# Prognostic Value of Volume-Based Parameters Measured by SSTR PET/CT in Neuroendocrine Tumors: A Systematic Review and Meta-Analysis

**DOI:** 10.3389/fmed.2021.771912

**Published:** 2021-11-26

**Authors:** Jiale Hou, Yi Yang, Na Chen, Dengming Chen, Shuo Hu

**Affiliations:** ^1^Department of Nuclear Medicine, Xiangya Hospital, Central South University, Changsha, China; ^2^Key Laboratory of Biological Nanotechnology, Changsha, China; ^3^National Clinical Research Center for Geriatric Disorders (XIANGYA), Xiangya Hospital, Central South University, Changsha, China

**Keywords:** positron emission tomography/CT, neuroendocrine tumors, somatostatin receptors, prognosis, tumor volume

## Abstract

**Purpose:** A meta-analysis was conducted to investigate the value of the volume parameters based on somatostatin receptor (SSTR)-positron emission tomography (PET) in predicting the prognosis in patients with neuroendocrine tumors (NETs).

**Material:** PUBMED, EMBASE, Cochrane library, and Web of Knowledge were searched from January 1990 to May 2021 for studies evaluating prognostic value of volume-based parameters of SSTR PET/CT in NETs. The terms used were “volume,” “positron emission tomography,” “neuroendocrine tumors,” and “somatostatin receptor.” Pooled hazard ratio (HR) values were calculated to assess the correlations between volumetric parameters, including total tumor volume (TTV) and total-lesion SSTR expression (TL-SSTR), with progression-free survival (PFS) and overall survival (OS). Heterogeneity and subgroup analysis were performed. Funnel plots, Begg's and Egger's test were used to assess possible underlying publication bias.

**Results:** Eight eligible studies involving 593 patients were included in the meta-analysis. In TTV, the pooled HRs of its prognostic value of PFS and OS were 2.24 (95% CI: 1.73–2.89; *P* < 0.00001) and 3.54 (95% CI, 1.77–7.09; *P* = 0.0004), respectively. In TL-SSTR, the pooled HR of the predictive value was 1.61 (95% CI, 0.48–5.44, *P* = 0.44) for PFS.

**Conclusion:** High TTV was associated with a worse prognosis for PFS and OS in with patients NETs. The TTV of SSTR PET is a potential objective prognosis predictor.

**Advanced in Knowledge**

The volume parameters based on SSTR PET can provide additional value for the prognosis of neuroendocrine tumors.

## Introduction

Neuroendocrine tumors (NETs) are a group of highly heterogeneous neoplasm originating from neuroendocrine cells and it can occur in different organs. The emergence of diagnostic technologies increases early-stage NETs and the detection rate of metastases, raising its incidence and prevalence (1). However, in patients with the same tumor stage and grade, the outcome of disease and survival of NET patients vary greatly (2, 3). Therefore, identifying the prognostic markers is crucial for the management of patients with NETs. Some studies showed that morphological imaging is of limited value in predicting the survival, disease progression, and treatment effects of NETs (4, 5). Several widely-studied diagnostic biomarkers, especially chromogranin A (CgA), has been widely studied. Its plasma level is affected by many factors including the use of proton pump inhibitors (6, 7), but its prognostic utility is still controversial (8, 9).

Somatostatin receptors (SSTRs) are expressed in most NET cells, particularly type 2, and is an ideal target for imaging and therapy method (10). SSTR-mediated imaging is considered to be more accurate than SSTR immunostaining in determining individual prognosis (11). SSTR PET imaging is considered a better imaging method than SSTR scintigraphy using ^111^In-octreotide due to its higher spatial resolution, higher image quality, and higher lesion detection rate (12). ^68^Ga-DOTA-peptides can be used to reflect the expression of SSTR, especially in well-differentiated NETs (WD-NETs). High maximum standardized uptake value (SUV_max_) is associated with a lower grade, better progression-free survival (PFS), and higher responsiveness to peptide receptor radionuclide therapy (PRRT) (13, 14). A meta-analysis by Lee and Kim (15) showed that the SUV_max_ of ^68^Ga-SSA is an important prognostic parameter for NETs patients. Low SUV_max_ is associated with the high risk of disease progression and mortality.

However, SUV_max_ reflects the value of a single voxel but does not represent the entire tumor. The volume parameters derived from PET in predicting the prognoses and monitoring the treatment can directly estimate systemic tumor burden, such as metabolic tumor volume (MTV) and total disease glycolysis (TLG), based on 2-deoxy-2-(^18^F) fluoro-D-glucose (^18^F-FDG) (16–19). However, well-differentiated NETs (WD-NETs) do not usually show high ^18^F-FDG uptake (20). SSTR-based PET/CT may be suitable for predicting the prognosis of WD-NETs patients. However, there are conflicting results regarding the prognostic value of volumetric parameters based on SSTR-PET in NETs (21, 22).

Therefore, we performed this meta-analysis to analyze the predictive value of volumetric parameters based on SSTR-PET for survival outcome in patients with NETs.

## Materials and Methods

The preferred reporting items for systematic reviews and meta-analyses (PRISMA statement) guidelines were used to perform this meta-analysis (23).

### Data Search and Study Selection

We performed a systematic search of PUBMED (to May 2021), EMBASE (to May 2021), Web of Science (to May 2021), and Cochrane (to May 2021) for English publications. The terms were as follows: (“neuroendocrine tumors” or “neuroendocrine tumor” or “tumor neuroendocrine” or “tumors neuroendocrine” or “neuroendocrine”) and (“PET”) or (“positron emission tomography”) and (“somatostatin receptor” or “SSTR”) and (“volume” or “volume-based parameters” or “tumor burden” or “tumor volume” or “volumetrical parameter”) and (“prognos^*^” or “predict^*^” or “Survival” or “outcome” or “PFS” or “OS” or “progress free survival” or “overall survival”). All searches were limited to human studies.

The inclusion criteria were studies using SSTR-based PET as an imaging tool, including volumetric parameters [total tumor volume (TTV) or total-lesion SSTR expression (TL-SSTR)] for whole body lesions and reported survival data. Reviews, abstracts, case reports, and editorial materials were excluded. Two authors independently searched and screened the eligible articles. A consensus resolved any discrepancies.

### Data Extraction and Quality Assessment

Data were extracted from the enrolled studies independently by two reviewers and the following information was recorded: first author, publication year, country, patient number, tumor grade, tumor site, radiotracer used, treatment after PET/CT scans, reported survival, PET volumetric parameters, and cut-off values of volumetric parameters.

Two reviewers independently used the quality in prognostic studies (QUIPS) tool to evaluate the quality of the included studies (24). The tool assesses the risk of bias in six domains including study participation, study attrition, measurement of prognostic factors, measurement of outcome, study confounding, and statistical analysis and reporting. Consensus was reached through discussion.

### Statistical Analysis

The primary outcome was PFS, including disease-free survival, recurrence-free survival, and event-free survival as the main outcome, and also the time interval from the date of starting therapy to the date of recurrence or metastasis. The secondary endpoint was overall survival (OS), defined as the time interval from the start of therapy to death from any cause. The effect of TTV or TL-SSTR on PFS and OS was measured by the effect size of the hazard ratio (HR). PFS or OS data were extracted using methods suggested in previous research (25). Univariate HR and 95% confidence intervals (CI) were extracted for each study, if provided by the author. If not, we used the Engauge Digitizer (http://markummitchell.github.io/engauge-digitizer/) to determine the survival rate according to the Kaplan–Meier curve to reconstruct HR estimate and its variance, assuming that patients were censored at a constant rate during the follow-up. Heterogeneity between studies was assessed by χ^2^ test and *I*^2^ statistics described by Higgins et al. (26). When *I*^2^ ≤ 50% and Cochran Q was *P* ≥ 0.1, a fixed effects model was used; when *I*^2^ > 50% or Cochran Q is *P* < 0.1, the random effect model was used. Subgroup analyses were performed according to the tumor grade and type of radiotracer. Further, funnel plots Begg's and Egger's test were performed to assess for any publication bias (27). Meanwhile, we performed the sensitivity analysis for prognosis by omitting each study to assess the influence of an individual study on the whole meta-analysis. *P*-values < 0.05 were considered statistically significant. Data from each study were analyzed using Review Manager (RevMan, Version 5.3; The Nordic Cochrane Centre, Copenhagen, Denmark) and Stata Version 15.0 (College Station, TX).

## Results

### Study Characteristics

**Figure 1:**
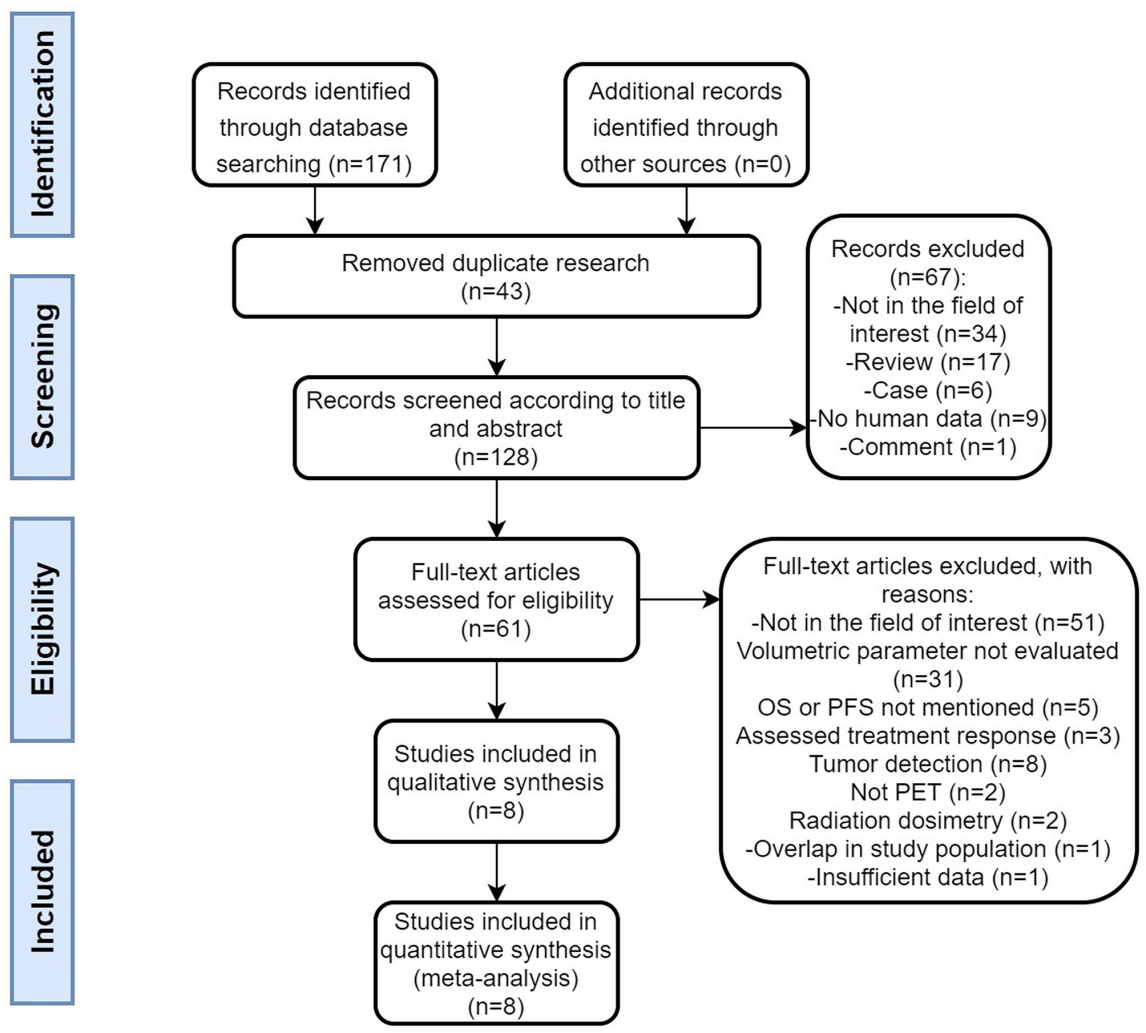
Flow chart.

A flow chart of the data search and selection is presented in Figure 1. A total of eight studies involving 593 patients were included in our meta-analysis. Five studies (21, 22, 28–30) were retrospective and three studies (31–33) were a prospective design. According to the WHO grade, three researches (22, 29, 30) included well-differentiated NETs (grade 1 and/or 2). Three studies had heterogeneous populations containing all grades (21, 31, 33) and the remaining two studies did not clearly state the grade of the enrolled patients (28, 32). All the eight studies included pancreas origin NETs and seven studies enrolled gastric intestinal (GI) tract origin NETs, including the stomach or/and midgut or/and rectum (21, 22, 28, 29, 31–33). Seven studies had other site origin NETs such as the lung, extrahepatic biliary tract, adrenal, and cancer of unknown primary origin (21, 22, 28, 29, 31–33).

The characteristics of the included study are shown in Table 1. From them, four studies used ^68^Ga-DOTATATE (22, 29, 31, 32), three studies used ^68^Ga-DOTATOC (21, 28, 30), and one study (33) used ^64^Cu-DOTATATE for PET imaging. The parameters included TTV in eight studies and TL-SSTR in two studies (21, 29). Seven studies (21, 22, 29–33) analyzed the prognostic value of TTV regarding PFS, and three studies further evaluated the relationship between TTV and OS (or disease-specific mortality) (28, 31, 33). Four studies reported the relationship between PFS and TL-SSTR (21, 22, 28, 29). Six threshold methods were applied for the measurement of TTV and TL-SSTR of whole-body lesions (Table 1). Cutoff value of TTV ranged from 7 to 578 ml, and the cutoff value of TL-SSTR in PET in two studies were 778.5 and 4,852 ml, respectively.

**Table 1:** Characteristics of the included study.

**No**.	**Study**	**Year**	**Country**	**Study design**	**Patient No**.	**Tumor Grade**	**Tumor site**	**Radio-tracer**	**Treatment After 68Ga-SSTR PET**	**End Point**	**Studied PET Parameters**	**Tumor delineation**	**Cut off value**
1	Tirosh et al.	2018	USA	prospective	184	I–III	Pancreas GI tract CUP Lung	^68^Ga-DOTATATE	medical, PRRT, LDT, Surgery	PFS and Disease-specific mortality	Total TV	Adaptive threshold by visual inspection	7.0 ml 35.8 ml
2	Toriihara et al.	2019	USA	retrospective	92	I–II	Pancreas GI tract CUP	^68^Ga-DOTATATE	Surgery, somatostatin analog, LDT, Radiation, PRRT	PFS	Total TV and TL-SSTR	50% of SUV_max_	11.29 ml
3	Ohlendorf et al.	2019	Germany	retrospective	33	I–II	Pancreas GI tract CUP	^68^Ga-DOTATATE	PRRT	PFS	Total TV and TL-SSTR	40% of SUV_max_	140.8 ml 4,852 ml
4	Ohnona et al.	2019	France	retrospective	50	I–II	Pancreas	^68^Ga-DOTATOC	surgery, somatostatin analog, chemotherapy, targeted therapy, PRRT, local therapy of a single metastatic site.	PFS	Total TV	41% of SUV_max_	13.8 ml
5	Kim et al.	2020	Republic of Korea	retrospective	64	I–III	Pancreas GI tract CUP	^68^Ga-DOTATOC	somatostatin analog	PFS	Total TV and TL-SSTR	1.5*liver SUV_mean_ + 2*standard deviation	58.9 ml 778.5
6	Pauwels et al.	2020	Belgium	retrospective	57	(–)	GI tract Pancreas CUP Other	^68^Ga-DOTATOC	PRRT	PFS and OS	Total TV and TL-SSTR	Adaptive threshold by visual inspection	578 ml
7	Carlsen et al.	2021	Denmark	prospective	116	I–III	GI tract Pancreas Extrahepatic biliary tract Lung CUP	^64^Cu-DOTATATE	Surgery LDT, external radiation. Interferon, somatostatin analog, chemotherapy and/or PRRT.	PFS and OS	Total TV	1.5*liver SUV_mean_ + 2*standard deviation	54.9 ml
8	Ortega et al.	2021	Canada	prospective	96	(–)	GI Pancreas CUP Lung Adrenal	^68^Ga-DOTATATE	PRRT	PFS	Total TV	SUV_max_ of liver/spleen	(-)

*CUP indicates cancer of unknown primary; PRRT, peptide receptor- radionuclide therapy; LDT, liver-directed treatment; OS, overall survival; PFS, progress free survival; TTV, total tumor volume; TL-SSTR, total-lesion somatostatin receptors expression; SUV_max_, maximum standardized uptake value*.

### Quality Assessment

According to the QUIPS tool quality assessment results, four studies (22, 30, 31, 33) had a moderate risk selection bias because they did not report whether the study population was consecutively selected, and two studies (21, 30) had high selection bias due to the relatively small number of cases enrolled in the group. All included studies showed a low risk of attrition bias. Regarding the measurement of prognostic factors, four studies (21, 29–31) showed a higher risk of bias due to the dependence on the cutoff value of the data, while two studies showed a moderate risk of bias because it was not mentioned whether blinded-manner was used in the measurement. For outcome measurement, seven studies (21, 22, 28–32) showed a moderate risk of bias because it was not clear whether the outcome measurement was performed without prognostic factors or the method used for the outcome measurement was unclear.

Regarding confounding bias, two studies (29, 31) showed high risk due to the lack of multivariate analysis. One study (28) showed moderate risk because grade was not considered. In terms of statistical analysis, two studies (22, 32) showed a higher risk of bias because the study included all variables that might be affected by multicollinearity into the multiple regression. In general, the results of the QUIPS tool indicated that the overall quality of the included studies was moderate (Supplementary Table 1).

### Prognostic Value of TTV and TL-SSTR on PFS and OS

**Figure 2:**
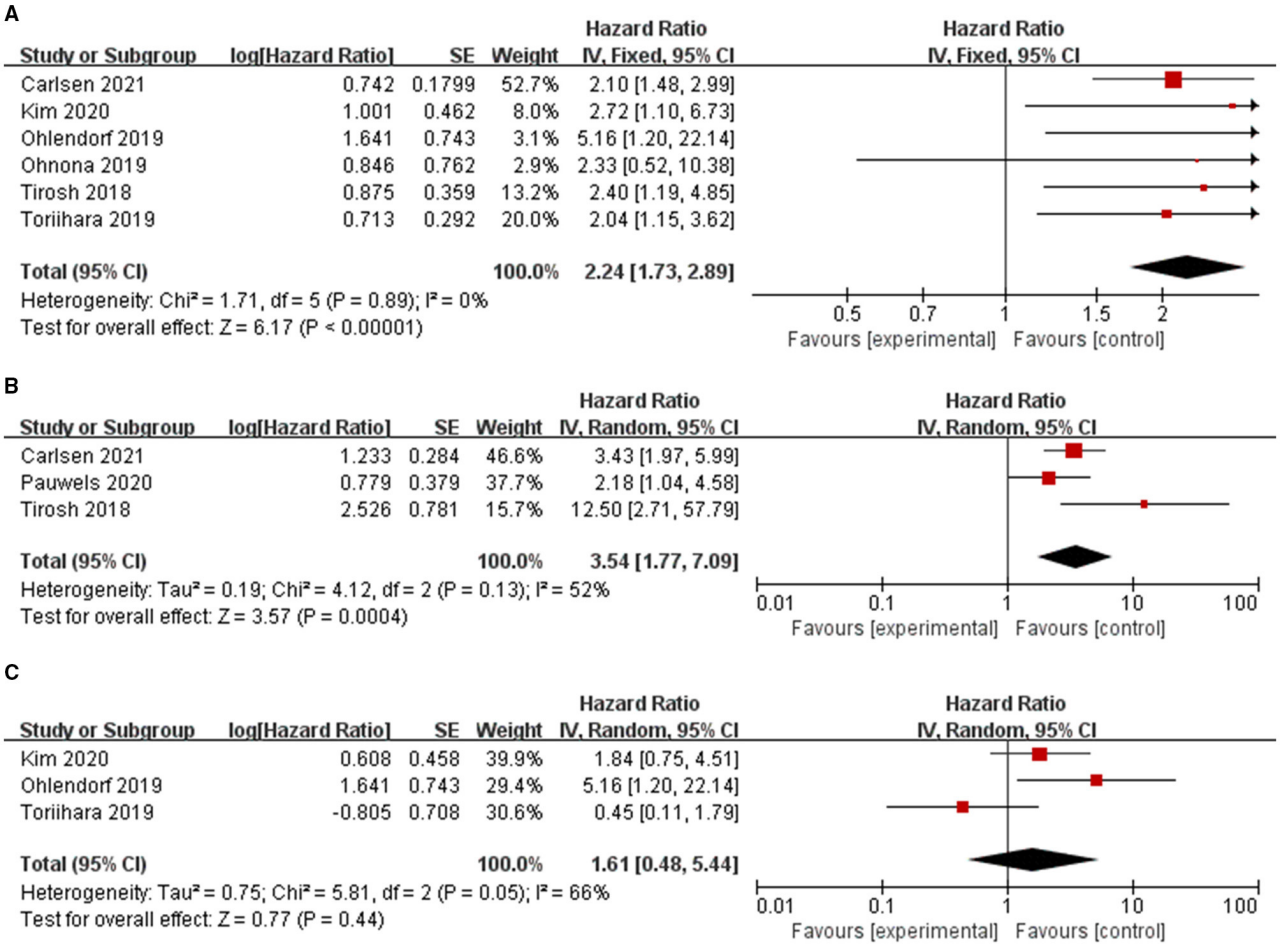
Forest plot results of the PFS **(A)** and OS **(B)** based on the total tumor volume and PFS based on the total tumor expressing SSTR **(C)**.

The effect of TTV on PFS was analyzed using seven studies. However, in one study (32), the study was omitted because HR could not be combined using continuous variables, while the other six studies were combined because all HR used binary variables. The combined HRs of 2.24 (95% CI: 1.73–2.89) was given a *I*^2^ of 0% using a fixed-model, showing a correlation between TTV and PFS (*P* < 0.00001) (Table 2; Figure 2). Also, we conducted sensitivity analysis (Supplementary Figure 1) to further estimate the impact on the combined HRs.

**Table 2:** Summary of the meta-analysis results.

**Parameter**	**Study no**.	**End point**	**HR**	**95%CI**	***P***	**Model**
TTV	6	PFS	2.24	1.73–2.89	<0.00001^*^	Fixed
TTV	3	OS	3.54	1.77–7.09	0.0004^*^	Random
TL-SSTR	3	PFS	1.61	0.48, 5.44	0.44	Random

*TTV, total tumor volume; TL-SSTR, total-lesion somatostatin receptors expression; HR, hazard ratio; CI: confidence intervals*.

* *Statistically significant (P < 0.05)*.

The effect of TTV on OS was analyzed using three studies. The combined HR was 3.54 with statistical significance (95% CI, 1.77–7.09; *P* = 0.0004). Heterogeneity was moderate (χ^2^ = 4.12, *P* = 0.13; *I*^2^ = 52%). The combined HRs were found to be stable, suggesting no individual study significantly affected the results (Supplementary Figure 1).

The effect of TL-SSTR on PFS was analyzed using three studies (21, 22, 29). A random-effects model was used and the pooled HR was 1.61 (95% CI, 0.48–5.44, *P* = 0.05; *I*^2^ = 66%, Figure 2; Table 2) with significant heterogeneity. The results showed no statistically significant correlations with PFS and TL-SSTR (*P* = 0.44).

### Subgroup Analysis

Subgroup analysis was performed to the tumor grade and type of radiotracer. Since the research on PFS based on TL-SSTR and the research on OS by TTV are relatively small, we only performed subgroup analysis on PFS based on TTV (Table 3). Among studies of TTV on PFS, no obvious heterogeneity was found between the studies on well-differentiated NETs (G1/2) (HR: 2.31, 95%CI: 1.40–3.82; *P* = 0.001) and studies on all grades of NETs (HR: 2.21, 95%CI: 1.64–2.98; *P* < 0.00001) (*I*^2^ = 0%, *P* = 0.88). Also there is no statistical difference between different imaging agents for predicting PFS (*I*^2^ = 0%, *P* = 0.88).

**Table 3:** Results of subgroup analysis in PFS based on TTV.

**End point**	**Factor**	**Study no**.	**Heterogeneity test (*I^**2**^*, *P*)**	**HR**	**95%CI of HR**	***P*-value**	**Model**
PFS	Well-differentiated NETs (G1, 2)	3	0%, 0.51	2.31	1.40-3.82	0.001^*^	Fixed
	All grades of NETs (G1–3)	3	0%, 0.85	2.21	1.64-2.98	<0.00001^*^	Fixed
PFS	^68^Ga-DOTATATE	3	0%, 0.51	2.34	1.53-3.58	<0.0001^*^	Fixed
	^68^Ga-DOTATOC	2	0%, 0.86	2.61	1.20-5.66	0.02^*^	Fixed
	^64^Cu-DOTATATE	1	NA	NA	NA	NA	NA

*NA, Not applicable*.

*PFS, progression-free survival; TTV, total tumor volume*.

* *Statistically significant (P < 0.05)*.

### Publication Bias

**Figure 3:**
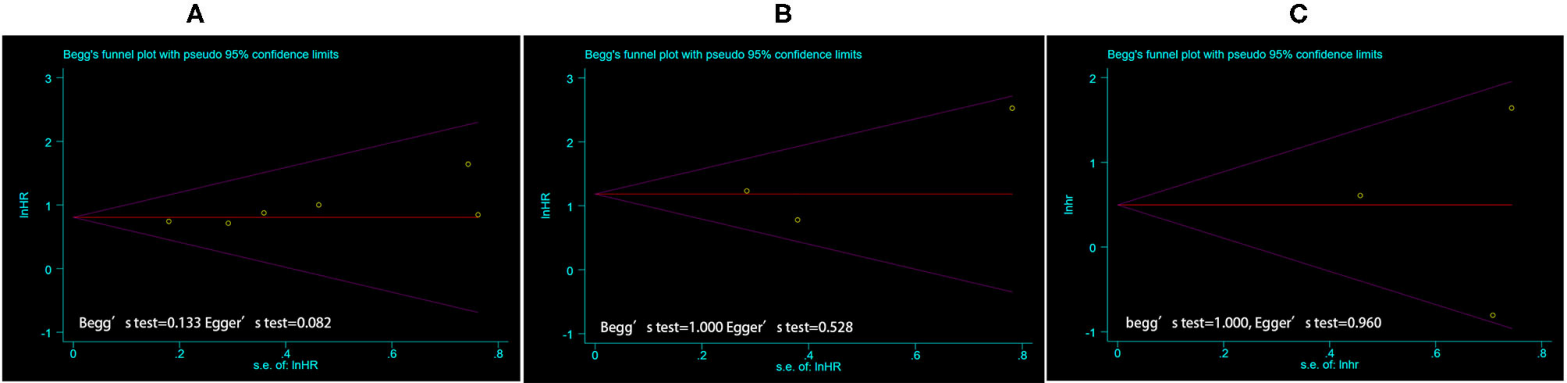
The funnel plot of publication bias estimates the results of PFS **(A)** and OS **(B)** based on TTV, and PFS based on TL-SSTR **(C)** in the meta-analysis. Egger's test and Begg's test were used for statistical analysis, where the *P* < 0.05 was considered as significant. PFS, Progress free survival; OS, overall survival; TTV, total tumor volume; TL-SSTR, total-lesion somatostatin receptors expression.

Begg's and Egger's tests were used to assess publication bias. The funnel plot and *P*-value estimation indicated no publication bias for TTV on PFS and OS, as well as for TL-SSTR on PFS (Figure 3).

## Discussion

To our knowledge, this is the first systematic review and meta-analysis to evaluate the prognostic value of volume-based parameters of SSTR PET/CT in NETs. The volumetric parameter based on SSTR PET is useful in predicting PFS. Subgroup analysis reveals that tumor grade and radiotracers may not affect the prognosis.

^18^F-FDG is the most common PET imaging agent, which can non-invasively assess tumor glucose metabolism and proliferation (34, 35). ^18^F-FDG PET can be used not only for diagnosis and staging, but also for assessing the proliferative activity and malignancy of tumors. Studies have shown that ^18^F-FDG may also reflect the prognosis of many tumors, including NET (36–38). A meta-analysis based on ^18^F-FDG PET/CT showed that MTV as a volumetric parameter of ^18^F-FDG PET may be an independent prognostic factor for survival (39). However, none of the studies we included had ^18^F-FDG PET volume parameters for predictive evaluation of prognosis. Although it is not clear whether volumetric parameters based on SSTR PET have better prognostic value than volumetric parameters based on FDG (MTV and TLG) in this study, tumor volume and total tumor expressing SSTR based on SSTR-PET as prognostic biomarkers of NETs have unique advantages compared with MTV or TLG. On the one hand, SSTR2 was an independent prognostic marker in NETs (11), and tumor volume based on SSTR was also correlated with PFS and OS (40). On the other hand, these SSTR-based volume parameters can better reflect the SSTR situation in entire tumors. In the future, we expect to directly compare the ability of ^18^F-FDG and SSTR PET parameters to predict prognosis through prospective studies.

In this review, higher TTV based on SSTR-PET showed shorter PFS and OS. Although the study of Ortega et al. (32) did not include the meta-analysis, the study still suggests that higher TTV is associated with a worse prognosis. Of six studies (21, 22, 28, 30, 32, 33) in which multivariate analysis for PFS was performed, four out of (22, 30, 32, 33) six were prognostic markers for PFS. Two out of (30, 32) three studies showed that the TTV were prognostic markers for OS. However, TL-SSTR was not significantly related to the prognosis in our study. Only Ohlendorf et al. (29) showed TL-SSTR was associated with PFS. The author believed that the difference may come from the different methods of tumor burden measurement and the samples of enrolled patients.

Heterogeneity was detected in this meta-analysis. In pooled data, significant heterogeneity was found for TTV based on SSTR-PET in predicting PFS. After excluding the study of Ortega et al. (32), the results of the overall estimated values aggregated by PFS reduced heterogeneity (*I*^2^, from 87 to 0%) with a HR of 2.24 (95% CI: 1.73–2.89). This may be due to the different tumor volume threshold, which should be discussed in a prospective study. Further analysis found that tumor grade revealed that the TTV of SSTR-PET could predict PFS and OS of all grades of NETs. Since the NET grade depends on the biopsy site, and the heterogeneity of NETs is high, the volume parameter may be more conducive to predicting the prognosis, but it still needs further research to confirm. Additionally, we also performed subgroup analysis of radiotracer types. Subgroup analysis found that the use of single ^68^Ga-DOTANOC and ^68^Ga-DOTATATE showed prognostic value. As we all know, ^68^Ga-DOTANOC, which binds specifically to sst_2_, sst_3_, and sst_5_ (41), has ten-times lower sst_2_ affinity than the sst_2_-selective tracer ^68^Ga-DOTATATE (42). A study has shown that ^68^Ga-DOTANOC performed better in detecting liver metastasis and had a higher tumor-to-background ratio in liver lesions due to the broader SSTR-binding profile (43). However, another study showed that ^68^Ga-DOTATATE detected more liver lesions, mainly due to a higher lesion uptake (44). Therefore, whether different radiotracers have a significant impact on the prognosis of tumor burden remains to be further studied.

To the best of our knowledge, this is the first meta-analysis to evaluate the prognostic value of volumetric parameters in the SSTR-PET in NETs. However, due to limited literature, it is difficult to directly compare the HRs between SUV_max_ and the volume parameter. In our study, volumetric parameters based on SSTR-PET were independent prognostic markers in three studies (29–31) of eight. SUV was found to be an independent prognostic marker in only one study (28) of eight.

This study has several limitations. Firstly, there were only three studies involving OS, and few studies involving TL-SSTR. Secondly, there were significant differences in study design, image analysis, cutoff value, sample sizes, and patient selection among the studies included in the current meta-analysis, leading to publication bias. Thirdly, due to the limited studies we enrolled, we cannot evaluate the best cut-off value of tumor burden parameters for prognostic prediction under the same primary site, treatment, and course of disease. We look forward to further research in future large-sample prospective studies.

## Conclusion

The TTV of SSTR-PET is a significant prognostic parameter in NETs patients. The high TTV is associated with an increased risk of disease progression and mortality, whether it is a well-differentiated NET group or a NET group of all grades. In the future, the TTV of SSTR-PET could be used as a potential predictor of prognosis in patients with NETs.

## Data Availability Statement

The original contributions presented in the study are included in the article/Supplementary Material, further inquiries can be directed to the corresponding author/s.

## Author Contributions

JH was responsible for experimental design, experimental analysis and thesis writing. YY and NC were responsible for literature retrieval, data screening, and article revision. DC and SH were responsible for the guidance and review of the thesis. All authors contributed to the article and approved the submitted version.

## Funding

This study was supported by the National Natural Science Foundation of China (Grant No. 91859207).

## Conflict of Interest

The authors declare that the research was conducted in the absence of any commercial or financial relationships that could be construed as a potential conflict of interest.

## Publisher's Note

All claims expressed in this article are solely those of the authors and do not necessarily represent those of their affiliated organizations, or those of the publisher, the editors and the reviewers. Any product that may be evaluated in this article, or claim that may be made by its manufacturer, is not guaranteed or endorsed by the publisher.
