# Supplementary material for: Prognostic Value of Volume-Based Parameters Measured by SSTR PET/CT in Neuroendocrine Tumors: A Systematic Review and Meta-Analysis
Source: Front Med (Lausanne). 2021 Nov 26;8:771912. doi: 10.3389/fmed.2021.771912 (PMC8662524; doi:10.3389/fmed.2021.771912)
Supplement: Supplementary file 1 [file Table_1.docx]

**Supplement Table 1. Summary of quality assessment of individual studies according to Quality in Prognostic Studies (QUIPS)**

| **Study** | **Year** | **1. Study Participation** | **2. Study Attrition** | **3. Prognostic Factor Measurement** | **4. Outcome Measurement** | **5. Study Confounding** | **6. Statistical Analysis and Reporting** |
| --- | --- | --- | --- | --- | --- | --- | --- |
| Tirosh, A. et al | 2018 | Moderate | Low | High | Moderate | High | Low |
| Toriihara, A. et al | 2019 | Moderate | Low | Low | Moderate | Low | High |
| Ohlendorf, F. et al | 2019 | High | Low | High | Moderate | High | Low |
| Ohnona, J. et al | 2019 | Moderate | Low | High | Moderate | Low | Low |
| Kim, Y. I. et al | 2020 | High | Low | High | Moderate | Low | Low |
| Pauwels, E. et al | 2020 | Low | Low | Moderate | Moderate | Moderate | Low |
| Carlsen, E. A. et al | 2021 | Moderate | Low | Low | Low | Low | Low |
| Ortega, C. et al | 2021 | Low | Low | Moderate | Moderate | Low | High |

**QUIPS risk of bias graph: The judgements about each risk of bias domain presented as percentages across all included studies (n=8).**
