# Supplementary material for: Prognostic Value of Volume-Based Parameters Measured by SSTR PET/CT in Neuroendocrine Tumors: A Systematic Review and Meta-Analysis
Source: Front Med (Lausanne). 2021 Nov 26;8:771912. doi: 10.3389/fmed.2021.771912 (PMC8662524; doi:10.3389/fmed.2021.771912)
Supplement: Supplementary file 3 [file Data_Sheet_2.docx]

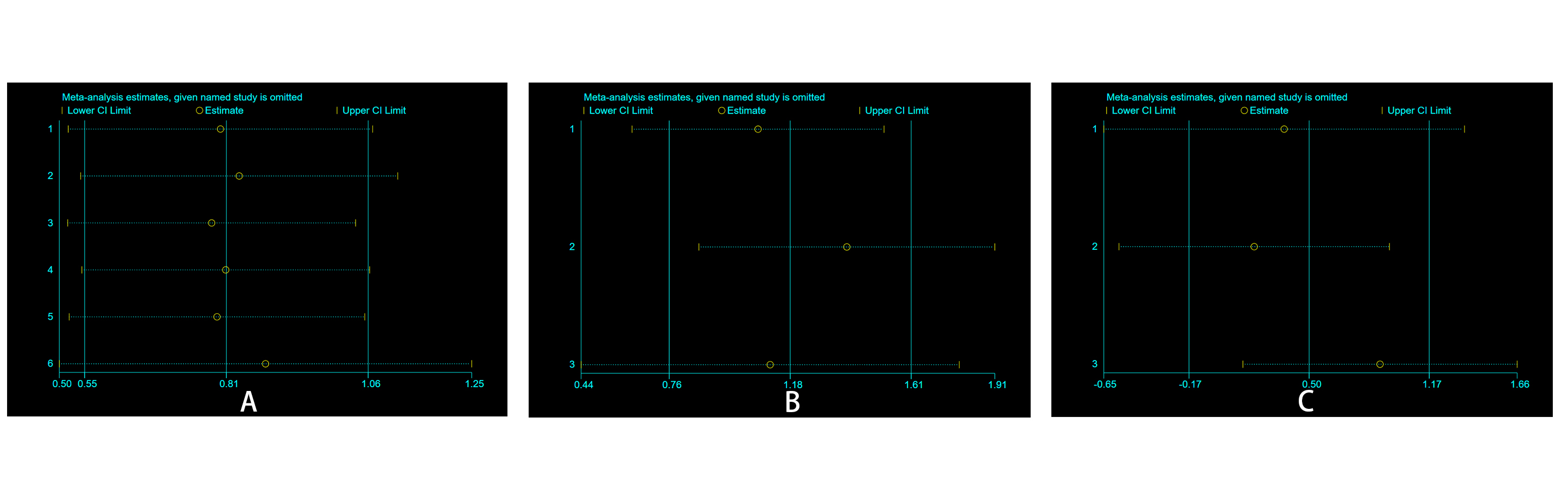


Supplement Fig. 1: Effect of individual studies on the combined HR, including TTV for PFS (A), the TTV for OS (B), and the TL-SSTR for PFS (C). HR: hazard ratio; TTV: total tumor volume; TL-SSTR: total-lesion somatostatin receptors expression OS: overall survival; PFS: progression-free survival.
